# Supplementary material for: Cross-cultural structures of personal name systems reflect general communicative principles
Source: Nat Commun. 2026 Jan 19;17:719. doi: 10.1038/s41467-025-67079-8 (PMC12819479; doi:10.1038/s41467-025-67079-8)
Supplement: Supplementary file 1 — Supplementary Information [file 41467_2025_67079_MOESM1_ESM.pdf]

Supplementary Information for: Cross-Cultural Structures Of Personal Name Systems Reflect General Communicative Principles

Michael Ramscar[a], Sihan Chen[b], Richard Futrell[c], Kyle Mahowald[d]

[a] *Tübingen University*
[b] *Massachusetts Institute of Technology*
[c] *University of California, Irvine*
[d] *The University of Texas at Austin*

Supplementary Note 1: Bootstrapping the correlations analysis in Finnish data

In Experiment 2, we calculated the Finnish prefix-name entropy of each birth year bin and each parish from a sample of 50 births, as the number of births in each parish varied. We found a main effect of birth year on prefix-name entropy, a main effect of longitude on prefix-name entropy, and an interaction between birth year and longitude on prefix-name entropy. Our results in Table 3 showed positive correlations between prefix-name entropy and the percentage of people having patronyms, as well as a positive correlation between prefix-name entropy and longitude, and such positive correlations decreased over time.

An anonymous reviewer raised an issue on the reliability of such a subsampling approach in calculating entropy. To address the reliability issue, we repeated the aforementioned analyses 500 times with different randomly sampled names and visualized the related statistical quantities as histograms in Supplementary Figure 1. First, the effects of birth year and longitude, as well as their interaction, remained robust, since among the 500 analyses, all of them showed a significant effect trending in the same direction (Supplementary Figure 1a) as was shown in Experiment 2. Similarly, the correlations among patronym percentage, longitude, and prefix-name entropy remained robust (Supplementary Figure 1b), since among the 500 analyses, all of them showed similar trend as was shown in Experiment 2. We also repeated the analysis by sampling 100 names each time and obtained similar results (Supplementary Figure 2).

Therefore, the results we showed in Experiment 2 were unlikely to be caused by artifacts introduced when we subsampled our data for entropy calculation.

Supplementary Note 2: Licensing information of datasets used in this study

In this study, we mainly used data from publicly available sources. Below is a compilation of permissions from our data sources. Our use of datasets fully complies with the terms and conditions set by each data provider.

Social Security Administration The Social Security Administration allows data to be used by "researchers interested in naming trends".[1]

U.S. Census Bureau The U.S. Census Bureau explicitly states that their datasets are made available for use in research.[2]

Wikipedia Data from Wikipedia is available for re-use under a CC-BY-SA license, [3] which allows its data to be shared and adapted as long as appropriate credits are given.

Taiwanese Ministry of Interior The Taiwanese goverment allows their published data to be freely used for noncommercial purposes.[4]

Korean Statistical Information Service One function of the Korean National Statistic Office is to make statistical data available for researchers.[5]

Population Data UK Population Data UK is a site "dedicated to providing information about the population of the United Kingdom".[6]

Data from Douglas A. Galbi One purpose of such dataset, according to Galbi, is "to spur further analysis of given names".[7]

National Records of Scotland All contents in the National Records of Scotland operates are available under the Open Government License v3.0, which allows its data to be copied, published, distributed, and transmitted as long as the source is acknowledged.[8]

HisKi database Permission to use the HisKi database has been given to several researchers. Several papers have been published using information in this database [e.g. 2, 3].

[1] `https://www.ssa.gov/oact/babynames/limits.html`

[2] `https://www.census.gov/data/developers/about/terms-of-service.html`

[3] `https://en.wikipedia.org/wiki/Wikipedia:Text_of_the_Creative_Commons_Attribution-ShareAlike_3.0_Unported_License`

[4] `https://data.gov.tw/license`

[5] `https://kostat.go.kr/menu.es?mid=a20602030000`. See the Dissemination of Statistical Information paragraph.

[6] `populationdata.org.uk`

[7] `https://www.galbithink.org/names/`

[8] `https://www.nationalarchives.gov.uk/doc/open-government-licence/version/3/`

References

1. R Core Team. *R: A Language and Environment for Statistical Computing* `http://www.R-project.org/` (R Foundation for Statistical Computing, Vienna, Austria, 2014).
2. Malmi, E., Gionis, A. & Solin, A. *Computationally inferred genealogical networks uncover long-term trends in assortative mating* in *Proceedings of the 2018 World Wide Web Conference* (2018), 883–892.
3. Kirielle, N., Christen, P. & Ranbaduge, T. *Outlier detection based accurate geocoding of historical addresses* in *Data Mining: 17th Australasian Conference, AusDM 2019, Adelaide, SA, Australia, December 2–5, 2019, Proceedings 17* (2019), 41–53.

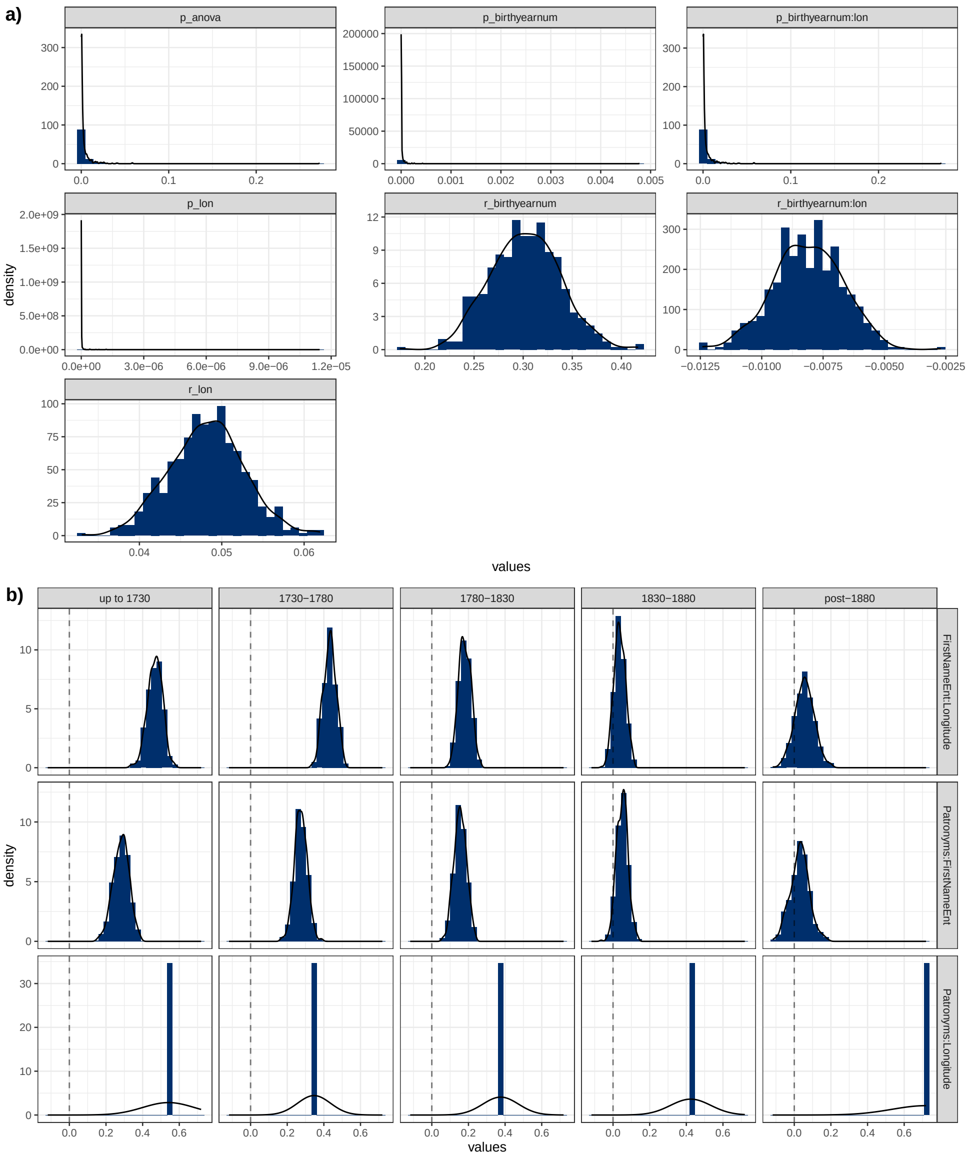

Supplementary Figure 1: **Results in Experiment 2 were independent of sampling**. Results from the bootstrapping analysis, where we sampled 50 births in each Finnish parish in each birth year bin, conducted the analyses in Experiment 2, and repeated the whole process for 500 times. **(a)** The distribution of two-sided p_values of model comparison (an interaction model vs. an additive model), two-sided p_values and the main effect of birth year on prefix-name entropy, two-sided p_values and the interaction between birth year and longitude, along with two-sided p_values and the main effect of longitude on prefix-name entropy. **(b)** The distribution of correlations between prefix-name entropy and longitude (first row), percentage patronym and prefix-name entropy (second row), and the percentage patrobym and longitude (third row), in each birth year bin (columns). The black curve in each graph represents the kernel density generated by `geom_smooth` function in R [1]

.

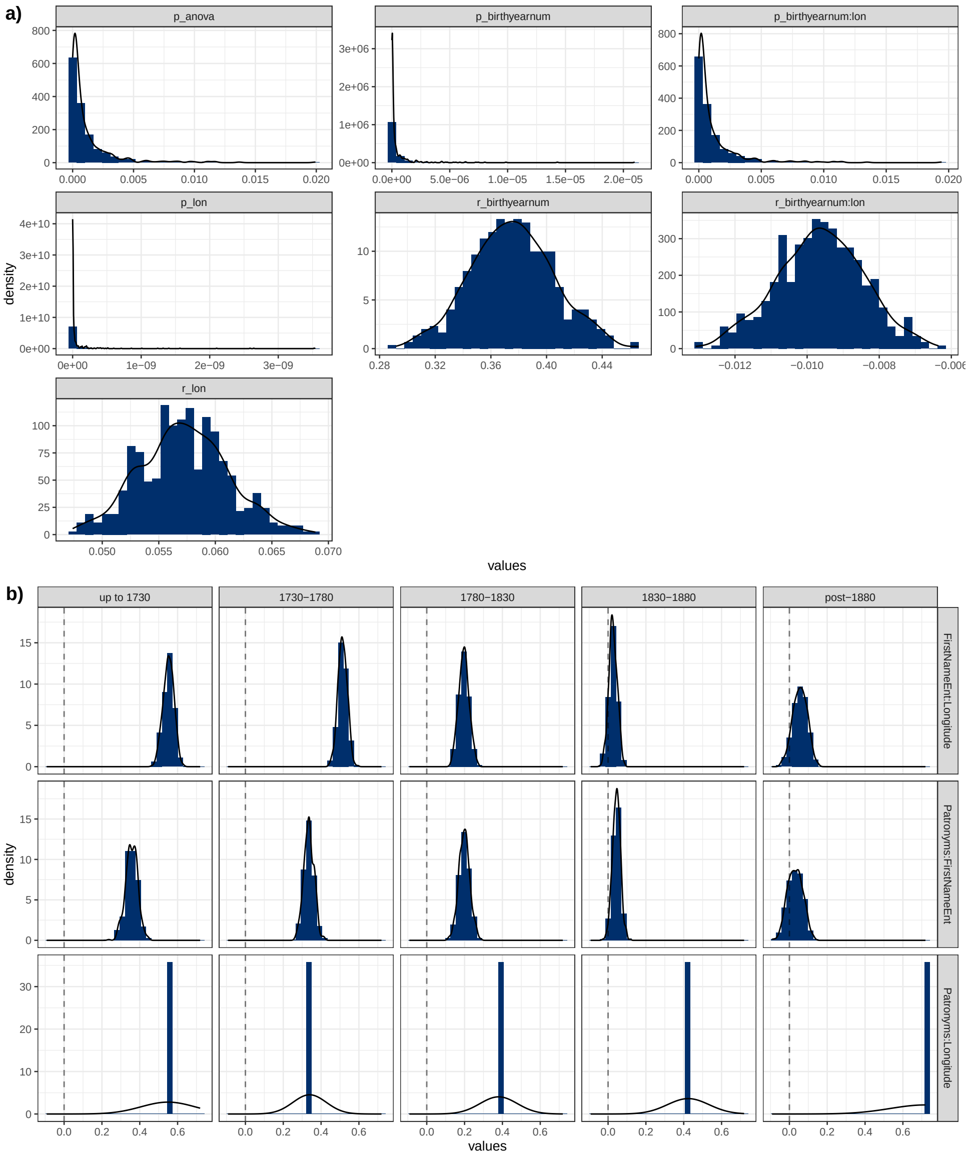

Supplementary Figure 2: **Results in Experiment 2 were independent of sampling**. Results from the bootstrapping analysis in which we sampled 100 births instead of 50 (as in Figure 1) in each Finnish parish in each birth year bin. The black curve in each graph represents the kernel density generated by `geom_smooth` function in R [1]

.
